# Compact Optical Nerve Cuff Electrode for Simultaneous Neural Activity Monitoring and Optogenetic Stimulation of Peripheral Nerves

**DOI:** 10.1038/s41598-018-33695-2

**Published:** 2018-10-23

**Authors:** Kang-Il Song, Sunghee Estelle Park, Seul Lee, Hyungmin Kim, Soo Hyun Lee, Inchan Youn

**Affiliations:** 1 Biomedical Research Institute, Korea Institute of Science and Technology, Hwarangno 14-gil 5, Seongbuk-gu, Seoul 02792 Republic of Korea; 2 Department of Bioengineering, University of Pennsylvania, Philadelphia, PA 19104 USA; 3 Department of Dentistry, Graduate School, Kyunghee University, 26, Kyungheedae-ro, Dongdaemun-gu, Seoul 02447 Republic of Korea; 4 Brain Science Institute, Korea Institute of Science and Technology, Hwarangno 14-gil 5, Seongbuk-gu, Seoul 02792 Republic of Korea; 5 KHU-KIST Department of Converging Science and Technology, Kyung Hee University, Seoul, 02447 Republic of Korea

## Abstract

Optogenetic stimulation of the peripheral nervous system is a novel approach to motor control, somatosensory transduction, and pain processing. Various optical stimulation tools have been developed for optogenetic stimulation using optical fibers and light-emitting diodes positioned on the peripheral nerve. However, these tools require additional sensors to monitor the limb or muscle status. We present herein a novel optical nerve cuff electrode that uses a single cuff electrode to conduct to simultaneously monitor neural activity and optogenetic stimulation of the peripheral nerve. The proposed optical nerve cuff electrode is designed with a polydimethylsiloxane substrate, on which electrodes can be positioned to record neural activity. We confirm that the illumination intensity and the electrical properties of the optical nerve cuff electrode are suitable for optical stimulation with simultaneous neural activity monitoring in Thy1::ChR2 transgenic mice. With the proposed electrode, the limb status is monitored with continuous streaming signals during the optical stimulation of anesthetized and moving animals. In conclusion, this optical nerve cuff electrode provides a new optical modulation tool for peripheral nervous system studies.

## Introduction

Over the last few decades, nerve cuff electrodes have been investigated for their use in neural signal recording and neuromodulation in peripheral nervous system (PNS) studies with minimally invasive and chronic implantation^1^. One of the purposes of using nerve cuff electrodes for the peripheral nerve is to monitor the physiology of the neuromuscular system with chronic implantation. For example, the axonal conduction velocity was measured to identify the order of motor neuron recruitment in a walking cat^2^, the cutaneous afferent activity was determined to investigate corrections in grip force^3^ and the long-term effects of axotomy were researched via the changes in neural activity measured through cuff electrode recordings after peripheral nerves were cut and sutured at their distal end to permit regeneration^4^. Nerve cuff electrodes can also be used for neuromodulation through electrical stimulation to restore motor function, for instance, when muscle recruitment is controlled for joint positioning^5^, when irregular neuronal activity is blocked to relieve pain^6^ or when the axonal conduction velocity is blocked to control urethral pressure^7^.

Optogenetic stimulation was introduced in recent studies as an alternative approach for neuromodulation. In this approach, specific cells are transfected with light-responsive targeted opsin^8,9^. The optogenetic stimulation of the peripheral nerve is an alternative solution to overcome the limitations of electrical stimulation, such as muscle fatigue and the reversed recruitment order of motor units^10^. For optogenetic stimulation of the peripheral nerves, a tethered optical fiber cable or light-emitting diode (LED) arrays were implanted on the targeted nerve to activate opsins that had been transgenically expressed or delivered through gene therapy. For example, the optical fiber was attached to the polydimethylsiloxane (PDMS) structure^11^, a micro-LED array was attached to the outside of a glass capillary pipette^10^ and a single micro-LED was positioned above the sciatic nerve using wireless power transmission^12^. This series of studies using animal-based experiments showed that optically induced neuromodulation of the peripheral nerve is an effective approach.

Despite the fact that diverse optical stimulation tools have been suggested for the neuromodulation of the PNS, the approach for monitoring neural activity during optogenetic stimulation remains insufficient. The neural signals from the peripheral nerve can provide information on sensory or motor organs, which can be interpreted to determine limb status using a neural decoding process^13,14^. For example, different initial joint positions and passive joint movements were estimated from muscle afferents recorded in the sciatic nerve^15,16^. The joint movements induced by electrical stimulation were also estimated from multichannel neural signals from the sciatic nerve^17^. With regard to the implantation feasibility, performing neural monitoring near the stimulation site is desirable to minimize invasiveness. A previous study related to monitoring the stimulation response, intramuscular activities, and muscle forces in anchored muscle tendons succeeded in verifying the response to optogenetic stimulation^10,18^. Although standard approaches for verifying the stimulation responses were used to record intramuscular activity and muscle force measurements in anchored muscle tendons, several electrodes had to be implanted to measure each type of muscle or to separate the muscle tendons to anchor the force transducer. Thus, the previous approaches were limited to implementation in a moving animal model for measuring the limb status from the response to stimulation.

**Figure 1:**
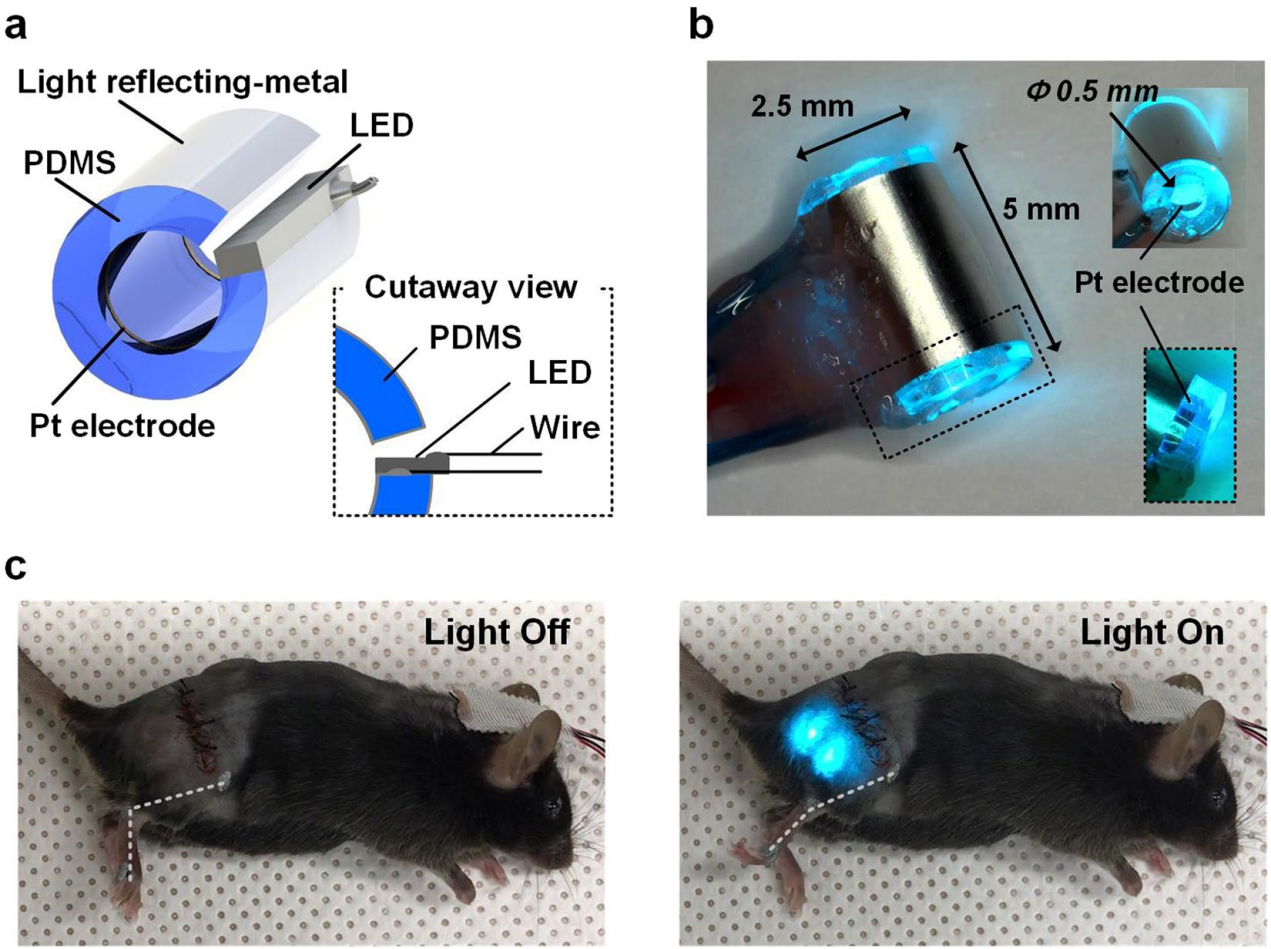
Optical nerve cuff electrode for optogenetic stimulation with simultaneous neural signal recording. (**a**) Overall schematic illustration of the optical nerve cuff electrode, (**b**) picture of an active device resulting in light illumination and (**c**) picture of an animal model for electrode implantation.

To address this consideration, we present a novel optical nerve cuff electrode for optogenetic stimulation with simultaneous monitoring of neural activity in the peripheral nerve. The proposed optical nerve cuff electrode comprises a PDMS substrate and neural activity recording electrodes (Fig. 1). This construction was suggested for simultaneous neural signal monitoring and optical stimulation within a single cuff electrode. The optical nerve cuff electrode was evaluated to verify that the illumination intensity was sufficient for performing optogenetic activation and for monitoring neural activity during optical stimulation. Next, the proposed optical nerve cuff electrode was implanted on the sciatic nerve in Thy1::ChR2 mice to achieve optogenetic stimulation with simultaneous neural signal recordings. In this study, we demonstrated the possibility of using the proposed optical nerve cuff electrode as an optogenetic tool for optical stimulation and neural activity monitoring in various PNS studies.

## Results

### Characteristics of the optical nerve cuff electrode

The optical nerve cuff electrode comprised a PDMS substrate for optical stimulation and electrodes for simultaneous monitoring of neural activity (Fig. 1a). The PDMS substrate was designed with a 3-mm diameter and 5-mm height, and its 0.5-mm inner diameter enables positioning around the sciatic nerve of mice. The PDMS substrate had a reflective metal sheet enabling the reflection of light back toward the nerve. For neural signal recording, a pair of electrodes was positioned on both sides of the PDMS substrate, which was connected to the neural signal amplifier system in a bipolar configuration.

**Figure 2:**
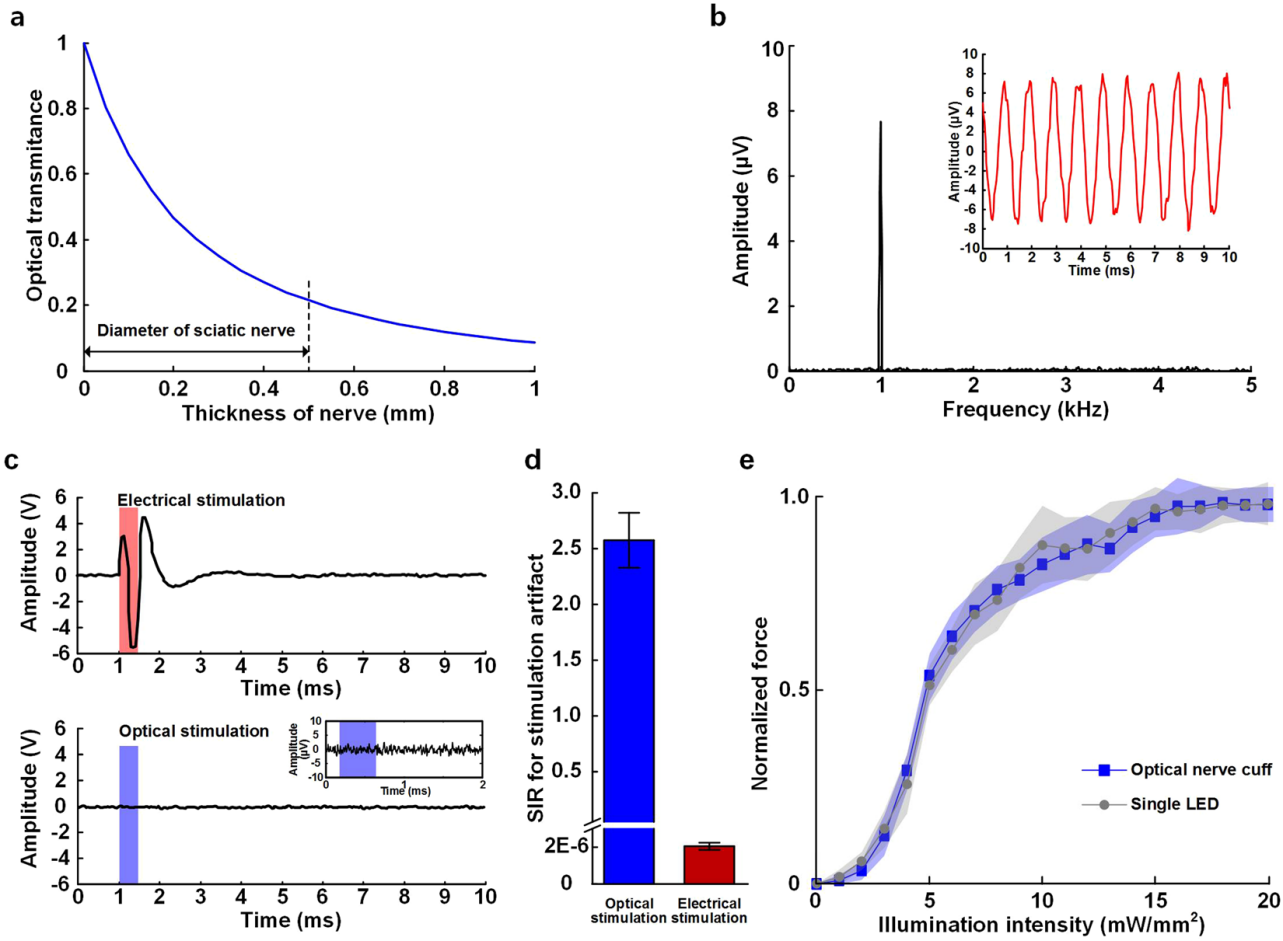
Illumination and electrical properties of simultaneous optical stimulation and neural signal monitoring using the optical nerve cuff electrode. (**a**) Estimated optical power transmission through the thickness of the sciatic nerve. (**b**) Neural signal recording with optical stimulation of the neural signal and the frequency domain analysis. (**c**) Waveform monitoring of the stimulation response during electrical and optical stimulations. (**d**) SIR of the stimulation artifacts during the electrical and optical stimulations. (**e**) Muscle force measurements during the optogenetic stimulation using the optical nerve cuff electrode and using a single LED.

Figure 2 shows the illumination and electrical properties of the simultaneous neural signal monitoring and optical stimulation using the optical nerve cuff electrode. The proposed optical nerve cuff electrode has the combined roles of optical stimulation and neural signal monitoring within a single electrode. Thus, we verified that the optical illumination power of the optical nerve cuff electrode was suitable for optogenetic stimulation of mice and that the electrical properties enabled recording of neural signals. The estimated optical power transmission through the thickness of the sciatic nerve is shown in Fig. 2a. The relative power (log(I/I_0_)) decreased from 1 to 0.24; the diameter of the mouse sciatic nerve was 0.5 mm. From the simulation results, we estimated that the initial light intensity of 5 mW/mm^2^ resulted in a light intensity of at least 2.05 mW/mm^2^ at the center of the nerve. In addition, a light intensity of at least 4 mW/mm^2^ at the nerve surface was required to transmit light to the end of the nerve and reach the minimum threshold for stimulating the Thy1::ChR2 mice. The neural signal recording using the optical nerve cuff electrode was verified in saline tank experiments (Supplementary Fig. S3). Figure 2c presents the results of the neural signal recordings. The recordings, which mimicked the neural signals as sine waves of 10 μV amplitude and 1 kHz frequency, were obtained in a manner similar to that for the simulated neural signals. The recorded signal was measured as sine waves of an 8 μV amplitude. A 1 kHz frequency was utilized for calculations by taking the Fourier transform and for verification of the characteristics of the frequency of the recorded signals. Figure 2d shows the results from monitoring the waveforms produced in response to produce to electrical and optical stimulations. An optical stimulation artifact could not be seen in continuous signal monitoring when comparing the optically stimulated signal to the electrically stimulated signal. However, in the electrical stimulation plot, stimulation artifacts were observed during electrical stimulation periods. The signal-to-interference ratio (SIR) of the stimulation artifact was calculated. For the SIR calculation, the signal is defined as the neural activity elicited by optical or electrical stimulation, and the interference noise is the stimulation artifact. The SIR for electrical stimulation (2e-6 ± 1.9e-7) was much less than that for optical stimulation (2.57 ± 0.24) (Fig. 2d). The muscle force trace was verified to be similar between measurements with the optical nerve cuff electrode and measurement with a single LED (Fig. 2e). The excitation threshold was lower with the single LED than with the optical nerve cuff.

### Optical nerve cuff modulation of motor neuron activity

**Figure 3:**
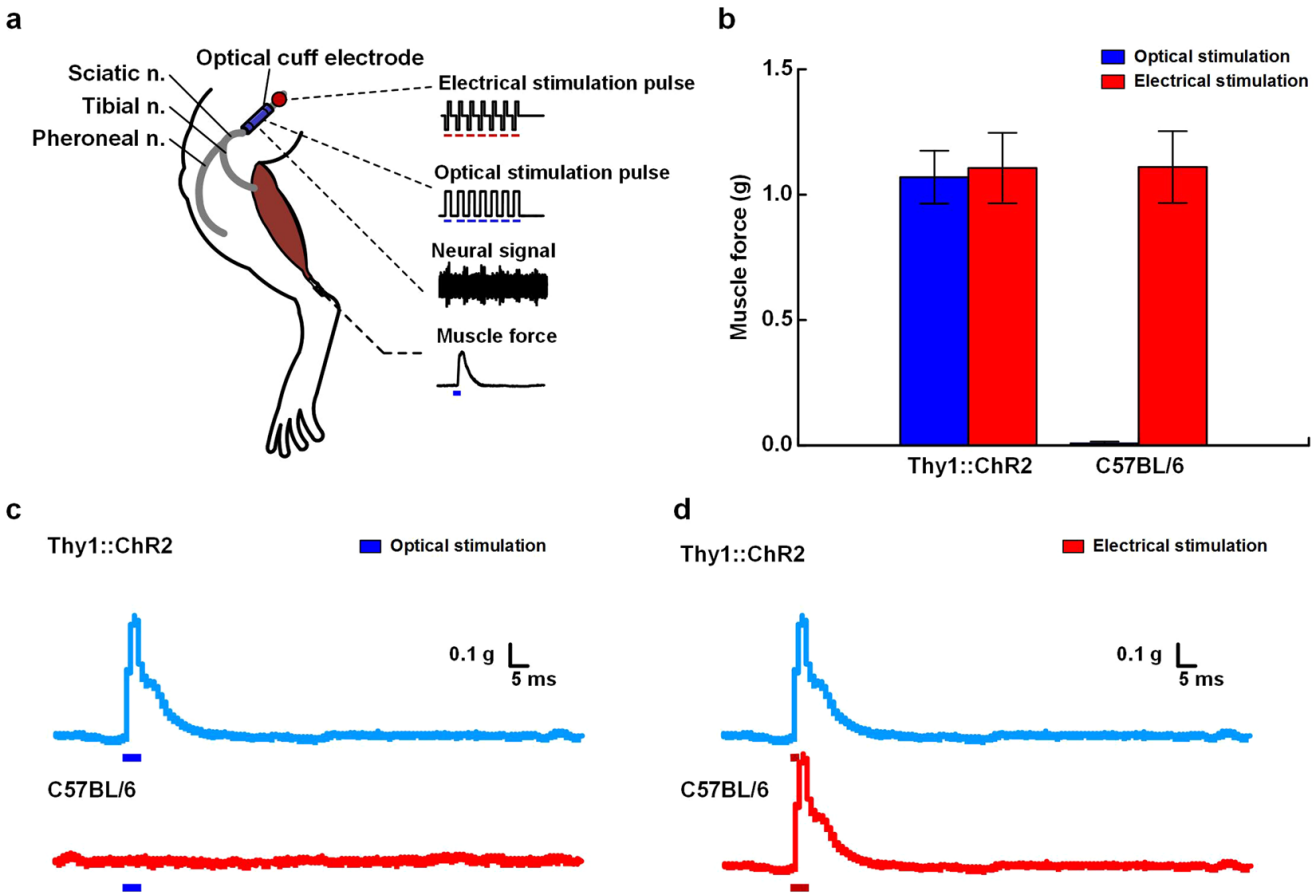
Effects of neural activity monitoring and muscle force measurements during optical and electrical stimulations. (**a**) Experimental setup for the simultaneous recording of neural activity during the optical and electrical stimulations. (**b**) Change in the muscle force during the optical and electrical stimulations in Thy1::ChR2 and C57BL/6 mice. Electrical stimulation pulse (biphasic pulses; pulse duration: 100 μs; current amplitude: 150 μA; and repeated frequency: 20 Hz) and optical stimulation pulse (pulse duration: 5 ms; illumination intensity: 10 mW/mm^2^; and repeated frequency: 20 Hz). (**c**,**d**) Typical force traces in Thy1::ChR2 and C57BL/6 mice during the optical and electrical stimulations. A single pulse stimulation is induced by either electrical (pulse duration: 100 μs; current amplitude: 150 μA) or optical (pulse duration: 5 ms; illumination intensity: 10 mW/mm^2^) stimulation.

We verified that muscle contraction was elicited by the optical modulation using the optical nerve cuff electrode (Fig. 3). An experiment was conducted such that each optical and electrical stimulation was applied to Thy1::ChR2 transgenic and C57BL/6 mice (Fig. 3a). During electrical stimulation, muscle force was elicited in both Thy1::ChR2 and C57BL/6 mice. The muscle force in the Thy1::ChR2 mice were 1.07 g ± 0.11 and 1.1 g ± 0.14 in response to optical and electrical stimulations, respectively. In addition, the muscle forces in the C57BL/6 mice were 0.004 g ± 0.004 and 1.11 g ± 0.14 in response to optical and electrical stimulation, respectively (Fig. 3b). The force traces are from twitches that were elicited by the optical and electrical stimulation of the Thy1::ChR2 and C57BL/6 mice (Fig. 3c,d).

### Simultaneous optical modulation of motor neurons and neural activity monitoring

**Figure 4:**
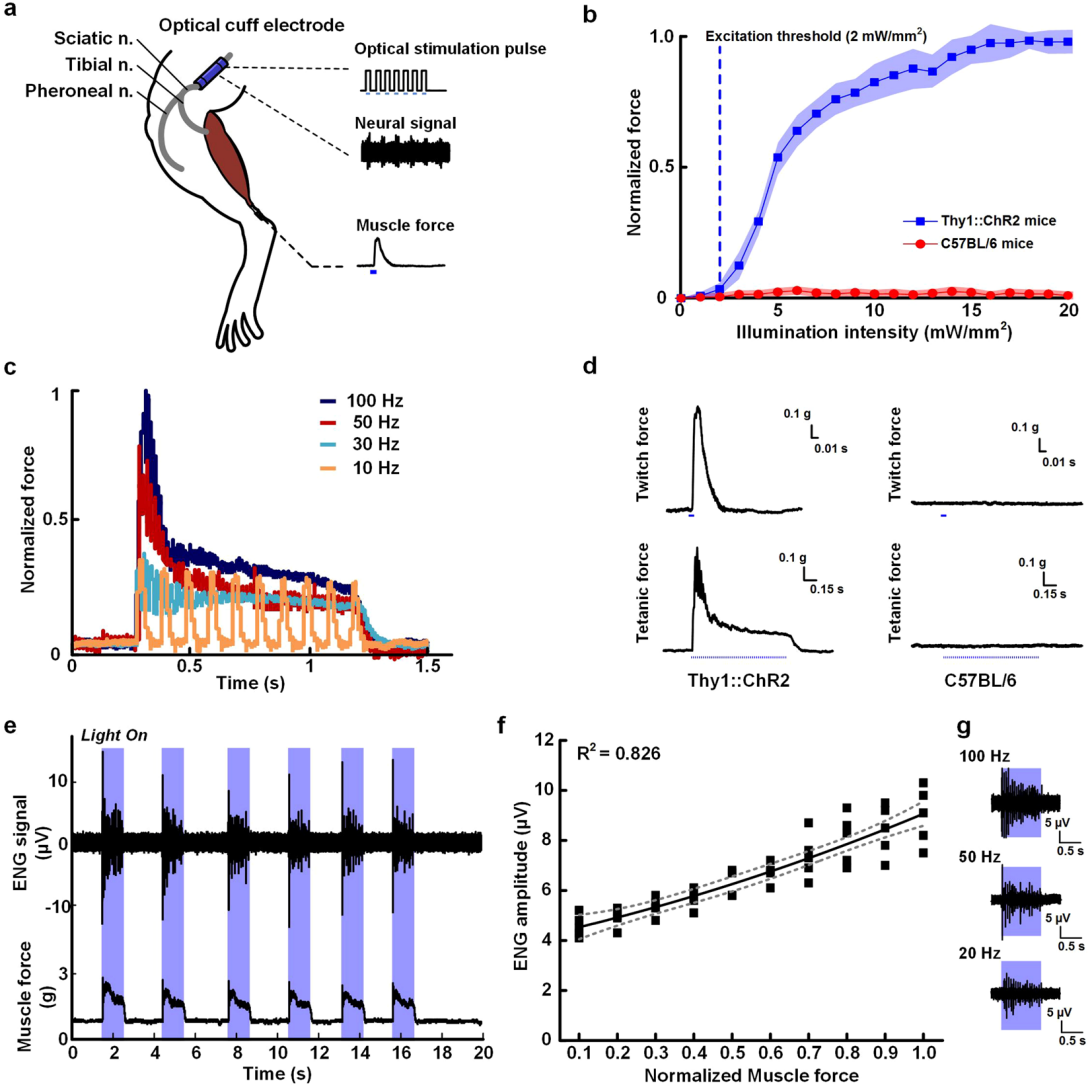
Optical controls of the simultaneous neural activity monitoring and optogenetic stimulation of the sciatic nerve of mice. (**a**) Schematic overview of the experimental setup for the optical control and neural activity monitoring of anesthetized mice. (**b**) Muscle force measurements during the optogenetic stimulation of Thy1::ChR2 and C57BL/6 mice. (**c**) Muscle force measurements with different stimulation frequencies (10 Hz, 30 Hz, 50 Hz and 100 Hz). (**d**) Typical force traces from the twitch and tetanic movements elicited by the optical stimulation. (**e**) Simultaneous neural activity monitoring during the optogenetic stimulation. The blue bars indicate the optical stimulation periods. (**f**) Correlation between the normalized muscle force and the neural signal amplitude. The correlation coefficient is R^2^ = 0.826. The gray dashed line indicates the 95% confidence interval. (**g**) Neural signal recording at different stimulation frequencies (20, 50, and 100 Hz). The blue bars indicate the optical stimulation periods.

Figure 4 illustrates the optical modulation of peripheral nerves with simultaneous monitoring of neural activity. The muscle force during the simultaneous monitoring of neural activity was measured to verify the optical control using the proposed optical nerve cuff electrode (Fig. 4a). The results of muscle contraction through variable illumination intensity are shown in Fig. 4b. An increase in normalized muscle force was shown above 2 mW/mm^2^ during optical stimulation in Thy1::ChR2 mice. The normalized muscle force became saturated by illumination intensities above 13 mW/mm^2^. In contrast, the normalized muscle force in the C57BL/6 mice was not influenced by the optical stimulation through the proposed electrode.

Figure 4c presents the changes in the muscle force with the frequency modulation. Changes in the normalized muscle force were detected at stimulation frequencies of 10 Hz, 30 Hz, 50 Hz and 100 Hz with a pulse duration of 5 ms and illumination power of 5 mW. The muscle force with the at 100-Hz stimulation frequency showed a large amplitude and tetanic force, whereas that at the 10-Hz stimulation frequency was smaller than those of the other frequency-modulated optical stimulations and the continuous tetanic force. In addition, the both twitch and tetanic muscle contractions were recorded during optical stimulation at 30 Hz. The muscle force became stronger as the frequency-modulated optical stimulation increased. In addition, the muscle forces were 0.98 g ± 0.03 and 0.01 g ± 0.02 in the Thy1::ChR2 and C57BL/6 mice, respectively. A force trajectory was observed only in the Thy1::ChR2 mice (Fig. 4d).

The possibility of simultaneous neural signal recording and optical stimulation is demonstrated in Fig. 4e. A neural signal was recorded during the frequency-modulated optical stimulation. The neural signal from the sciatic nerve during an optical stimulation of 1s slightly decreased following the decrease in the muscle forces. We also investigated the correlation between the muscle force and the neural signal amplitude. The muscle force increased when the neural signal increased with an *R*^2^ value of 0.826 (Fig. 4f). The neural signal density and amplitude increased more at the 100-Hz stimulation frequency than at the 20-Hz stimulation frequency (Fig. 4g).

**Figure 5:**
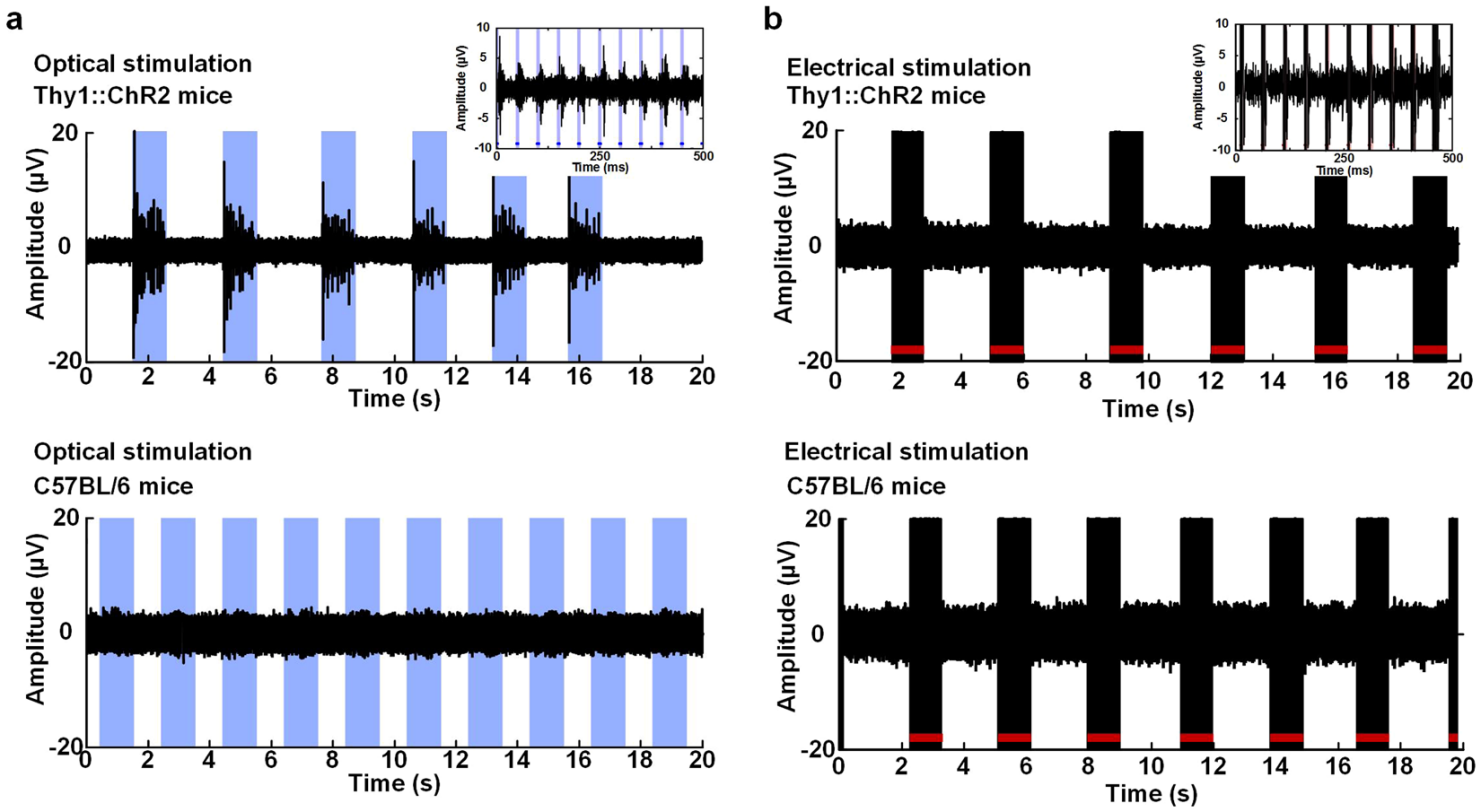
Neural activity monitoring in Thy1::ChR2 and C57BL/6 mice. (**a**) Neural activity monitoring during optical stimulation. (**b**) Neural activity monitoring during electrical stimulation. The blue bars indicate the optical stimulation periods. The red bars indicate the electrical stimulation periods.

**Figure 6:**
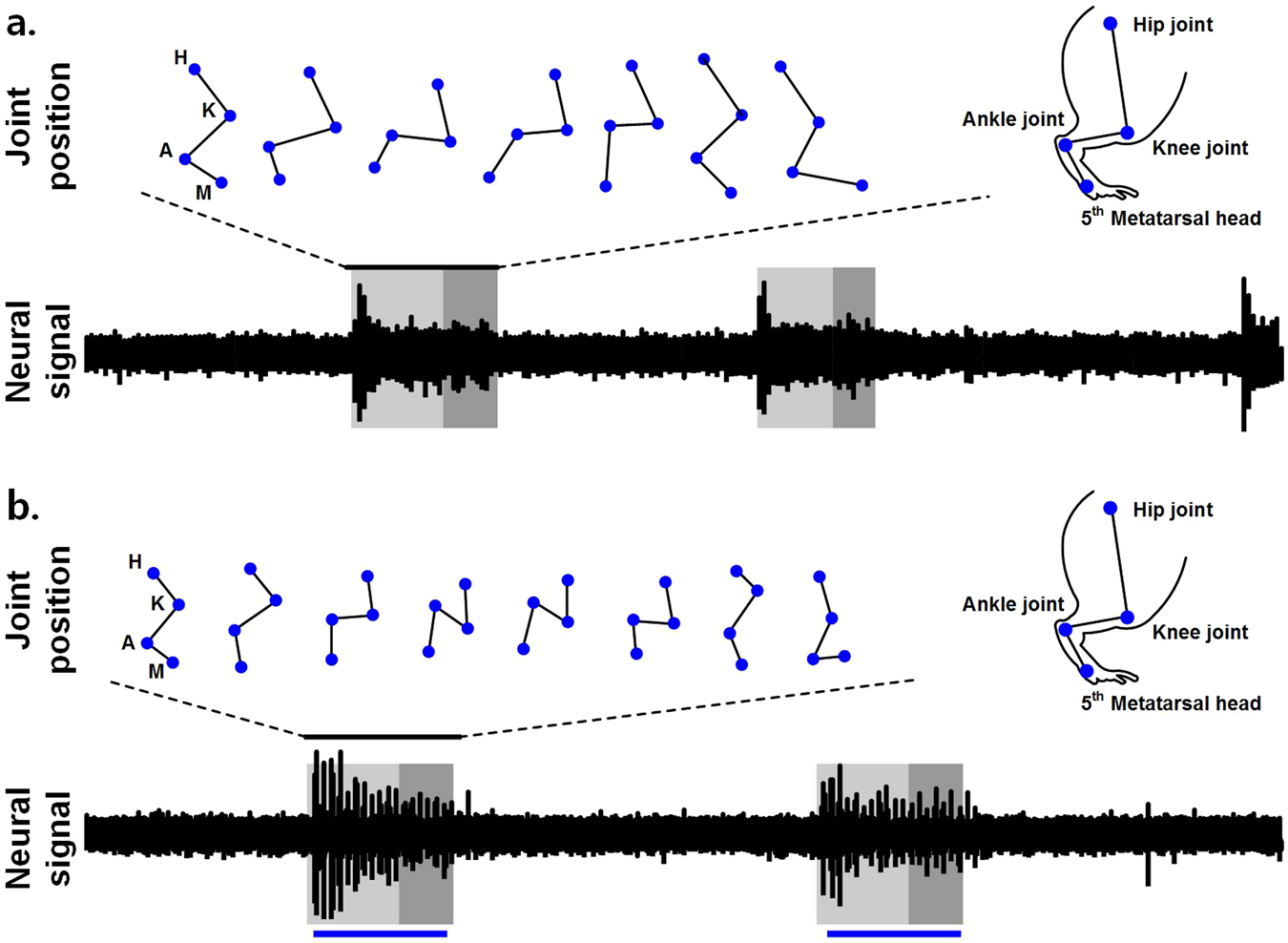
Typical examples of the simultaneous neural activity monitoring and optical stimulation of moving animals. (**a**) Neural activity monitoring in a moving animal without optical stimulation. (**b**) Neural activity monitoring in moving animals with optical stimulation. The black bars denote the neural signal periods during a single gait cycle. The blue bars denote the optical stimulation periods.

The neural signals in the stimulation artifact during the simultaneous neural signal recording and optical stimulation using the proposed optical nerve cuff electrode were robust. The stimulation artifact had contaminated neural signals when electrical stimulation induced the neural signal recording. In contrast, the neural signal recording with the optical stimulation was free from the effects of the electrical stimulation artifact (Fig. 5). Furthermore, we investigated the neural activity with optical stimulation in moving animals (Fig. 6). The joint position during the gait cycle was separated into the stance and swing phases. The stance phase was defined as the period from the initial contact to the toe-off position during the gait cycle, while the swing phase was the period from the toe-off position to the next initial contact. The two peak extensions of the neural signal were observed at the transition between the initial contact and the toe-off position during one gait cycle (Fig. 6a). In contrast, the neural signal in the neural signal recording with optogenetic stimulation showed several peaks, and the ankle joint was contracted to the dorsi-flexion position during the optical stimulation periods (Fig. 6b).

### Temperature changes in the sciatic nerve from optical nerve cuff modulation

**Figure 7:**
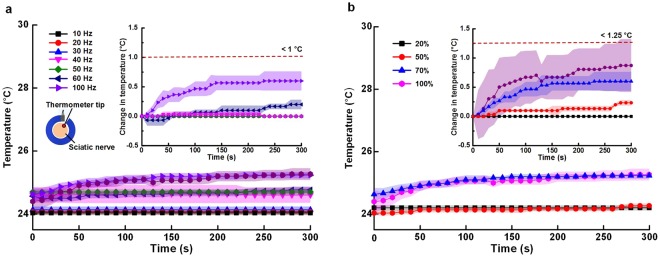
Effects of thermal damage on the nerve tissue during optical stimulation at different illumination intensities. (**a**) Temperature changes during optical stimulation at different stimulation frequencies. (**b**) Temperature changes during optical stimulation at different stimulation duty cycles.

We verified that temperature changes in the sciatic nerve from optical modulation using optical cuff electrodes (Fig. 7). In Fig. 7a, the temperature changes are shown within 1 °C during the stimulation periods of 300 s with the different stimulation frequencies (i.e., 100 Hz, 60 Hz, 50 Hz, 40 Hz, 30 Hz, 20 Hz, and 10 Hz). The lowest temperature changes were lower than 0.5 °C at the stimulation frequency under 100 Hz. In Fig. 7b, the temperature changes showed similar results in different stimulation periods with duty cycle variation; the maximum temperature variation was within 1.25 °C at the 100% duty cycle.

### Histochemical analysis

**Figure 8:**
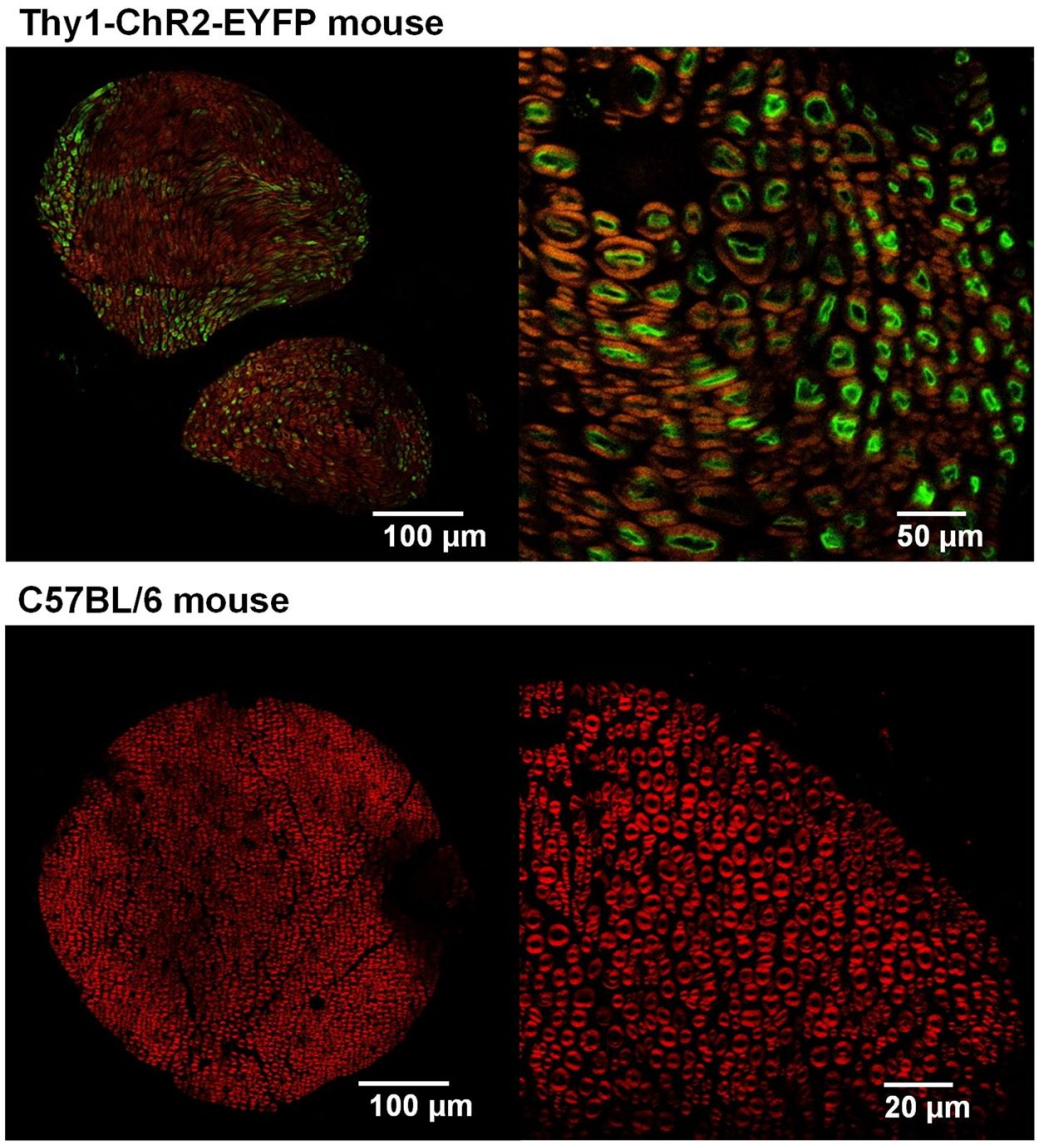
ChR2 expression in the sciatic nerve of a Thy1::ChR2 mouse. Confocal image of the sciatic nerve in a Thy1::ChR2 mouse and a C57BL/6 mouse. Motor neurons expressing *ChR2-EYFP* (green) and FluoroMyelin (red). Myelin (red) surrounds the neurons (green) in the Thy1::ChR2 mouse.

The Thy1::ChR2 expression in the motor neurons in the sciatic nerves of the Thy1::ChR2 mice was verified using confocal images (Fig. 8). The confocal image shows that the ChR2-EYFP-expressing axons in the green part of the figure are surrounded by myelin sheaths with a red fluorescent myelin label in the Thy1::ChR2 mice. The ChR2::EYFP expression level was sufficiently high, which was proven by the fact that all axons expressing ChR2::EYFP in the nerves of the transgenic mice were brighter than the background levels observed in the axons of the C57BL/6 mice.

## Discussion

In this study, a novel optical nerve cuff electrode was proposed for the simultaneous optical stimulation and neural activity monitoring of Thy1::ChR2 mice. Previous works developed optogenetic stimulation tools for PNS modulation using a micro-LED to fabricate a miniature and implantable device^10,12^. Although these optogenetic stimulation tools were successful in achieving practical PNS neuromodulation, these tools focused only on the optical stimulation for neuromodulation. In contrast, our proposed optical nerve cuff electrode was designed to achieve simultaneous neural activity monitoring and optical stimulation with a single electrode. One advantage of the proposed system is that it can be used to monitor activity during optical stimulation. Neural signals can be used as source signals to control the stimulation intensity, which can be used to determine the status of the muscle or to detect neurological disorders. Another advantage is that the number of electrodes for neural signal recording and stimulation can be reduced. Conventional approaches are limited by the need to implant the nerve cuff electrode in the narrow spaces of the distal branch of nerves^3,5^ and the separation required between each stimulation and recording electrode in the distal branch of nerves or muscles^6,14^. In addition, neural signal distortions occurred because of the lack of sufficient space when the stimulation and recording nerve cuff electrodes were implanted along the sciatic nerve. The proposed simultaneous optogenetic stimulation and neural activity monitoring using a single cuff electrode made it possible to achieve both optogenetic control and status monitoring and requires a narrow site for implantation in the peripheral nerves. Research on the PNS requires recording of the neural activity during the optogenetic stimulation to monitor the status of the stimulation conditions.

The key to miniaturizing these optical nerve cuff electrodes is the integration of a PDMS substrate for optical stimulation with neural signal-recording electrodes. As demonstrated with conventional nerve cuff electrodes, the substrate with the electrodes was wrapped around the nerve, providing a stable approach for monitoring the neural activity of the peripheral nerve^1,2^. The configuration of the PDMS substrate with the electrode yielded a miniaturized electrode capable of simultaneous neural activity monitoring and optogenetic stimulation (Fig. 1). Compared with the conventional optogenetic stimulation tools with an LED array^10^, the proposed optical cuff made it possible to use fewer LEDs and could be useful when constructing the miniaturized optical system. In the construction of the proposed optical nerve cuff electrode, the PDMS substrate was wrapped around the sciatic nerve, and the micro-LED was mounted on the PDMS substrate, which had an outer metal layer that could reflect light back toward the nerve. Recent neural interface devices have considered the use of a low-modulus elastomer, providing a lower mechanical mismatch with biological tissue, and PDMS can achieve a lower stiffness that is comfortable on the peripheral nerve^19^. The use of the PDMS substrate can result in lower mechanical mismatch between the implant and the peripheral nerve compared with previously developed optical cuff electrodes made with pipet glass.

The muscle force in the previous optogenetic stimulation studies was elicited with different illumination intensities^10,18^ and the increased illumination intensity led to an increased muscle force. Similar to the results of these studies, the muscle forces modulated by our proposed optical nerve cuff were elicited with different illumination parameters. The role of optical stimulation in optogenetics is to excite an optically sensitive gene with light of a specific wavelength^9^. In a previous work, Thy1::ChR2 opsin was activated with 473 nm light at an illumination intensity ≥1 mW/mm^2^. The results of the muscle force measurements performed at different illumination intensities (Fig. 4b) showed that the illumination intensity of the proposed optical nerve cuff electrode was adequate for a range of motor neuron excitations at intensities ≥2 mW/mm^2^ and led to saturated muscle forces at intensities ≥16 mW/mm^2^. The results of muscle contraction at different stimulation frequencies also confirmed that the proposed optical nerve cuff electrode was suitable for optogenetic control in peripheral nerves.

The advantage of the optical nerve cuff electrode in simultaneous neural signal monitoring and optogenetic stimulation was that the stimulation artifacts did not contaminate the neural signals (Fig. 4e). In neural signal recordings induced by functional electrical stimulation, a stimulation artifact appeared in the neural signals obtained using nerve cuff recording^6^. To obtain the pure neural signals, additional procedures for artifact removal were needed to use the neural signals contaminated by stimulation artifacts and to eliminate this interference because the amplitude of the stimulation artifacts was larger than that of the neural signals. One simple method for removing artifacts is the blanking process, which eliminates the stimulation artifacts that ground the neural signals during synchronized stimulation periods^17,20^. However, this process results in data loss during the stimulation periods. In contrast, the results of the simultaneous optical stimulation and neural activity monitoring using the proposed optical nerve cuff electrode showed that the neural signals recorded during the optical stimulation were free of stimulation artifacts. The investigations showed that using the optical nerve cuff electrode could obviate the additional artifact removal process and could measure the neural signal without data loss. Consequently, simultaneous neural monitoring and optical stimulation did not require additional sensors. The muscle force measurements involved highly invasive approaches using an ankle tendon-anchored transducer. Using the neural signal to alter the sensors to monitor the limb status may be possible based on the strong correlations between the muscle force and the neural signal amplitude during the optical stimulation (Fig. 4f). The neural signal recorded during the optical stimulation could be used to evaluate peripheral nerve diseases. The mechanism used for the optogenetic stimulation of the PNS could be utilized to apply additional neural processing.

The proposed optical nerve cuff electrode showed that the nerve tissue was free from thermal damage. The temperature changes during optical stimulation ranged from 24 °C to 26 °C to elicit joint movement. In addition, the temperature changed less than 1.25 °C at an illumination duty cycle of 100%. In the previous works by Xu^21^, nerve damage and neural signal blocking occurred due to local heat from the rat sciatic nerve, which was evident as the temperature reached 47 °C. Temperature changes due to optical illumination could not influence the nerve tissue damage and neural signal recordings.

Furthermore, the proposed optical nerve cuff electrode is applicable to the measurement of neural signals and to the optical stimulation of a moving animal. The result shows that simultaneously recording the neural signals and performing the optical stimulation with a single sensor may be possible. Additionally, since normal gait was observed from the joint position during the nooptical stimulation period, the proposed optical nerve cuff electrode was not obstructed during movement behavior. Lu^22^ recently presented an optogenetic tool that also employed simultaneous optical stimulation and neural monitoring for optogenetic stimulation. For application in the peripheral nerve for neural signal recording, the optical fiber should penetrate the peripheral nerve. However, by following the conventional neural signal methodology of the cuff electrode, the proposed optical nerve cuff electrode could be applied to the peripheral nerve without penetrating the nerve. This implementation was shown to be possible with various PNS tools for monitoring and stimulating peripheral nerves and for neurological disorders. In recent works, optogenetic stimulation tools were improved with consideration of the material properties of the soft tissue for long-term effects^22,23^. In further work, we could verify the long-term effect of the proposed optical neve cuff electrode to demonstrate its feasibility as a powerful tool for optogenetics-based long-term PNS studies.

In conclusion, the optical nerve cuff electrode was an effective PNS study tool because of its capability of simultaneous optical neuromodulation and neural activity monitoring. Integrating the recording and stimulation of electrodes to minimize electrode usage and to overcome the spatial limitations of electrode implantation was possible. Furthermore, the experiments with free movement showed that with our proposed system, the neural activity could be robustly monitored with optical control of the hind limbs of mice. The optical control with neural activity monitoring using the proposed novel optical nerve cuff electrode was useful for modulating and recording the PNS. In addition to its extensive applicability to PNS studies, our proposal has the potential for widespread use in research and in future clinical applications for optogenetic stimulation outside the brain.

## Methods

### Construction of the optical nerve cuff electrode

The optical nerve cuff electrode was constructed on a cylindrical waveguide substrate with platinum electrodes. The waveguide substrate was fabricated using polydimethylsiloxane (PDMS) (Sylgard 184 silicon elastomer, Dow Corning, USA). The PDMS was poured on a cylindrical PDMS mold and cured to obtain the cylindrical shape. The PDMS was mixed with a curing agent with a base polymer ratio of 1:10 before curing. Bubbles were then removed under vacuum. A rectangular aluminum sheet (4 mm wide, 10 mm long, and 0.05 mm thick) was embedded outside the waveguide substrate and covered with PDMS to reflect the optical light through the waveguide. After curing, the cylindrical PDMS substrate was cut to a 4.5 mm length with its edges overlapping by 0.25 mm. For the neural signal recording, two platinum electrodes were positioned on the inner sides of the cylindrical PDMS substrate. Each electrode was 0.5 mm wide and 2.5 mm long. A pair of electrodes was connected to the neural signal monitoring system in a bipolar configuration. A micro-LED with a wavelength of 473 nm (LXML-PB0-0040, Lumileds, Netherlands) was coupled to the edge of the side of the PDMS substrate perpendicularly and was bonded with additional PDMS (Fig. 1a). The interconnection for neural signal recording and optical stimulation was sealed with silicon (Esthetic mask, Detax, Germany). The schematic procedure is shown in the supplementary document (Supplementary Fig. S1).

### Light propagation simulation

The light propagation was modeled using the Kubelka–Munk model (modified by Aravanis *et al*.^24^ for diffuse scattering tissue) to verify the light propagation across all regions of the sciatic nerve. The light intensity varied with distance as follows:

$$\frac{{I}_{(z)}}{{I}_{(z=0)}}=\frac{{\rho }^{2}}{(Sz+1)(z+\rho )}$$

where,

$$\rho =r\sqrt{{(\frac{n}{NA})}^{2}-1}$$

*S* is the scattering length of the tissue, z is the distance of the light source, *r* is the diameter of the LED chip, and *n* is the refractive index of the material through which the light traverses. *NA* is the numerical aperture of the LED given by:

$$NA=n\,\sin \,{\theta }_{1/2},$$

where *θ*_*1/2*_ is the half angle of divergence from the LED. This model considered the effect of both scattering and geometrical losses on the light intensity. The value used for the scattering length was empirically determined from the mouse sciatic nerve for a wavelength of 473 nm based on the work by Michoud^23^. The light propagation simulation was assumed to have no losses in light intensity due to absorption. Moreover, it did not consider multiple scattered photons. The values for *θ*_*1/2*_ and *r* were taken from the data sheet provided by the LED manufacturer, whereas *I*_(Z=0)_ was measured with a power meter (PM100D, Thorlabs, USA).

### Animal preparation

All animal experiments were performed and handled in accordance with the regulations of the Institutional Animal Care and Use Committee of the Korea Institute of Science and Technology (Approval No. 2018-010). The experimental procedure was performed according to the Guide for the Care and Use of Laboratory Animals. Ten week-old adult male B6.Cg-Tg (Thy1-COP4/EYFP)18Gfng/J (25 g, Jackson Lab, USA) mice and C57BL/6 mice were used in this study. The mice were deeply anesthetized using a 3:1 cocktail of diluted Zoletil (140.9 mg of zolazepam mixed with 145.5 mg of tiletamine in 5 mL of sterilized injection solution, Virbac, France) and xylazine (23.32 mg/mL) at a final volume of 0.1 mL/100 g (intraperitoneal injection) to implant the optical nerve cuff electrode. The skin incision was extended to the dorsal aspect of the paw to expose the hind limb musculature. The biceps femoris and semitendinosus were then identified and retracted to expose the sciatic nerve. The optical nerve cuff electrode was wrapped around the sciatic nerve after the surrounding tissue was removed.

### Data acquisition

For neural signal recording, the cuff electrode system developed in a previous study^25^ was modified to accommodate the bipolar configuration recording. A preamplifier module and an external amplifier module with a gain of 39601 and a −3 dB bandwidth from 425 to 5500 Hz were connected during the neural signal recording. The muscle force was measured using a force transducer (72–4495, Harvard Apparatus, USA) attached to the distal tibialis anterior (TA) tendon secured with a suture wire. The hind limb was secured to the testing plate with Kirschner wires (Pfizer Howmedica, Rutherford, USA) through the femur and the ankle with the medial side down. The neural signal recording from the sciatic nerve, which was the final output of the external amplifier and the muscle force, was synchronously digitized using an analog-to-digital converter board (PXI-6733, National Instruments, USA) and a sampling frequency of 25 kHz.

For the neural signal recording of a moving animal, the preamplifier was positioned on the head of the mice with a transcutaneous head connector. The neural signal was obtained using an analog-to-digital converter board (PXI-6733, National Instruments, USA) and a sampling frequency of 25 kHz. Two-dimensional motion analysis was performed to measure the joint position of the hind limb. The motion analysis system consisted of a digital camera, a 2.5-mm-diameter reflector marker and an image grabber board with a 640 × 480 pixel resolution. The digital camera (Marlin F033B, AVT, Germany) was positioned perpendicular to the walking track, and the camera recorded images at a 60 fps sampling rate. Each joint position was marked on the skin over anatomical landmarks on the lateral side of the hind limb^26^. The neural signal and the joint position were synchronously recorded using a PXI-1042Q chassis (National Instruments).

The temperature data were measured from anesthetized mice for verifying thermal damage on the nerve tissue during optical stimulation. The thermometer tip was positioned in the gap between the sciatic nerve and the inner surface of the optical cuff electrode. The temperature data were recorded using a digital thermometer during illumination for a duration of 300 s each (repeated for a total of 3 measurements), with frequency variations (10, 20, 30, 40, 50, 60, 70, and 100 Hz) and duty cycle variations (25, 50, 70, and 100%). All data were analyzed from 3 mice.

### Optical and electrical stimulation

For the optical stimulation, a computer-controlled optical stimulation system developed in a previous study^27^ was modified to modulate the micro-LED. The light pulse duration in all experiments was set to 5 ms. The sciatic nerve was stimulated at a twitch frequency of 1 Hz. Five twitches were obtained at 20 different illumination intensities. During the tetanic trials, the sciatic nerve was stimulated at frequencies of 10, 30, 50, and 100 Hz with an illumination intensity of 10 mW/mm^2^.

For the electrical stimulation, the microelectrode (563410, A-M Systems, USA) was positioned on the sciatic nerve at a 1-mm distance above the optical nerve cuff electrode with an electrical stimulator (AM 2200, A-M Systems, USA) and computer control using LabVIEW (National Instruments, USA). A balanced biphasic pulse (pulse width of 200 μs and repeat frequency of 20 Hz) with amplitude modulation was induced on the sciatic nerve.

### Statistical analysis

*In vivo* peripheral nerve control for muscle contraction was performed in each leg of five animals. All data were then analyzed using one-way analysis of variance. The groups with varied light intensities and frequencies were compared pairwise using Tukey’s test to investigate the interrelationships between the groups. A value of p < 0.05 was considered statistically significant in all cases.

### Histology

The Thy1::ChR2 mice expressed *ChR2*, which was fused with an enhanced yellow fluorescent protein (*EYFP*) in the peripheral nervous system (PNS). First, the Thy1::ChR2 mice were anesthetized and perfused with 4% paraformaldehyde in phosphate-buffered saline (PBS). The sciatic nerves were then dissected, postfixed (overnight with 4% paraformaldehyde) and transferred to 30% sucrose in PBS, in which the mouse nerves were kept overnight. The sciatic nerves were embedded in Tissue-Tek OCT compound (Sakura Tissue-Tek, USA) and sectioned on a Leica CM1950 cryostat (Leica, USA). For immunohistochemistry, the sciatic nerve was cut in the longitudinal and cross-sectional directions at a 20-μm thickness, then mounted onto glass slides, and stained with FluoroMyelin (F34652, FluoroMyelin, ThermoFisher Scientific, USA). The slices were first rinsed in PBS and 0.2% Triton X-100 for 10 min and then permeabilized in PBS, 10% fetal bovine serum, and 0.2% Triton X-100 for 10 min. They were then treated with FluoroMyelin in PBS (1:300) for 30 min. As a final step, the nerve tissue was rinsed in PBS for 10 min and then mounted with Fluoroshield Mounting Medium (ab104135, Abcam, UK). The images were taken with a confocal microscope (TCS-SPE, Leica, USA).

## Electronic supplementary material

Supplementary Information
