# Supplementary material for: Compact Optical Nerve Cuff Electrode for Simultaneous Neural Activity Monitoring and Optogenetic Stimulation of Peripheral Nerves
Source: Sci Rep. 2018 Oct 23;8:15630. doi: 10.1038/s41598-018-33695-2 (PMC6199280; doi:10.1038/s41598-018-33695-2)
Supplement: Supplementary file 1 — Supplementary Information [file 41598_2018_33695_MOESM1_ESM.pdf]

Supplementary information

Compact Optical Nerve Cuff Electrode for Simultaneous Neural Activity Monitoring and Optogenetic Stimulation of Peripheral Nerves

Kang-Il Song[1], Sunghee Estelle Park[1,2], Seul Lee[3], Soo Hyun Lee[4], Inchan Youn[1,5,*]

[1]Biomedical Research Institute, Korea Institute of Science and Technology, Hwarangno 14-gil 5, Seongbuk-gu, Seoul 02792, Republic of Korea
[2]Department of Bioengineering, University of Pennsylvania, Philadelphia, PA 19104, USA
[3]Department of Dentistry, Graduate School, Kyunghee University, 26, Kyungheedae-ro, Dongdaemun-gu, Seoul 02447, Republic of Korea
[4]Brain Science Institute, Korea Institute of Science and Technology, Hwarangno 14-gil 5, Seongbuk-gu, Seoul 02792, Republic of Korea
[5]KHU-KIST Department of Converging Science and Technology, Kyung Hee University, Seoul 02447, Republic of Korea
[*]Corresponding author

1. Fabrication of the optical nerve cuff electrode

The optical nerve cuff electrode was fabricated following these procedures (Fig. S1): first, the PDMS mold was designed to construct the cylindrical flexible waveguide substrate. Subsequently, two platinum foil electrodes were placed on the metal rod (1 mm diameter, stainless steel) with approximately 2/3 of the circumference of the metal rod. The electrodes were positioned 4 mm apart (Fig. S1**a**). The platinum foil electrode used had a wire interconnected to preamplifier to record the neural activity.

Next, the metal rod was positioned on the PDMS mold in alignment with the central axis. PDMS was poured over the space between the metal rod and the PDMS mold and then cured at 90 °C for 3 h (Fig. S1**b**). Subsequently, the cured PDMS structure was released from the PDMS mold.

The light-reflecting metal was adhered to the outside of the cured PDMS structure and covered with a 0.01 mm PDMS layer. Subsequently, the cuff opening was manually created using a slit/cut on the cured structure with a 1 mm gap. A micro-LED was mounted on the side of the cured structure (Fig. S1**c**). Finally, the metal rod was carefully removed in an ultrasonic water bath.

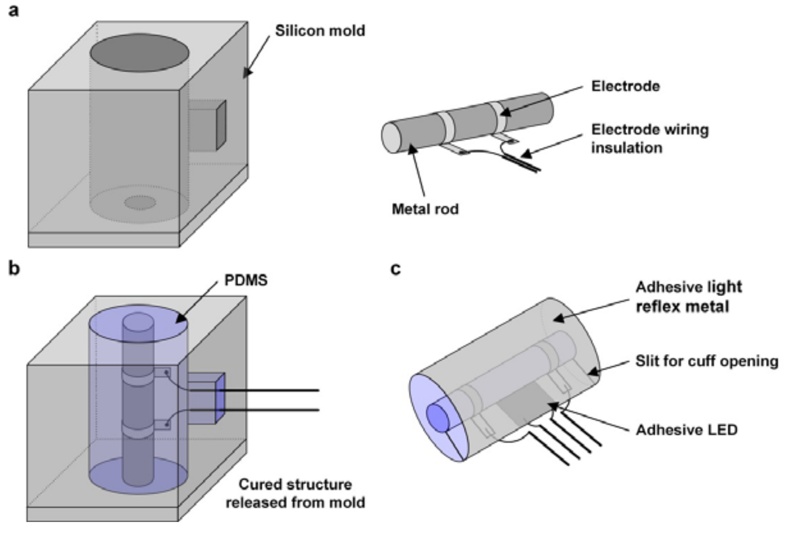

Fig. S1. Schematic of the fabrication of the optical nerve cuff electrode.

2. Electrical characteristics

Fig. S1 shows the variation in the impedance magnitude of the cuff electrode with frequency. The impedances of the cuff electrode were measured by a potentiostat (VersaSTAT4, Princeton Applied Research). The impedance spectroscopy measurements were performed at an open-circuit potential in the frequency range from 100 mHz to 100 kHz with an amplitude of 10 mV. An Ag/AgCl (in saturated KCl) electrode and a platinum wire were used as the reference and counter electrodes, respectively. The impedance was approximately 1 kΩ at 1 kHz in the frequency range of 300–5000 Hz.

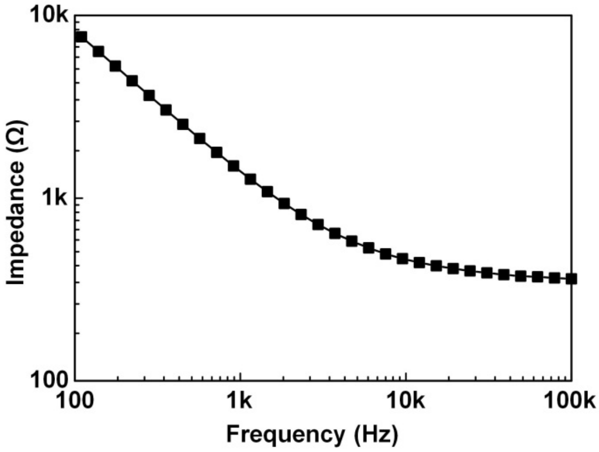

Fig. S2. Impedance magnitude of the optical nerve cuff electrode over frequency.

3. ***Verification of the optical nerve cuff electrode***

Saline tank experiments were performed to verify the performance of the optical nerve cuff electrode. These experiments were a modification of those from a previous study[1,2] (Fig. S3). The source generator representing the neural signal applied to two Pt electrodes inside the saline solution (0.9% NaCl) provided 1 kHz sinusoidal signals with a 10 μV peak. The optical nerve cuff electrode was also immersed in the saline solution and connected to a neural signal amplifier system modified from previous works[3]. The neural signal voltage was measured under optical stimulation to verify the performance of the simultaneous neural signal recording and optical stimulation. The light current provided 1 mA peak and 50 Hz square pulses to illuminate the optical source. The electrical stimulation provided 100 μA peak and 100 μs biphasic pulse through two Pt electrodes generated from the neural signal voltage. The effects on the neural signal recording between the optical and electrical stimulation were then compared.

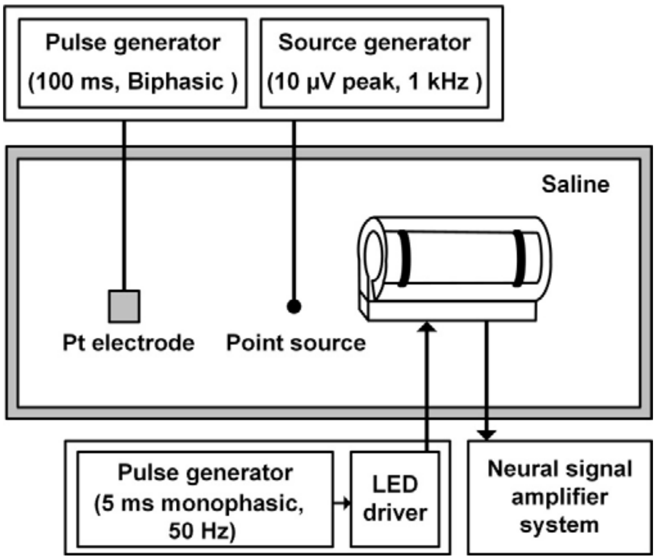

Fig. S3. Schematic overview of the saline tank experiments used to verify the electrical properties of the optical nerve cuff electrode.

4. ***Thermal damage assessment of sciatic nerve***

A histological assessment was performed to verify the thermal damage of the sciatic nerve during optical stimulation. Hematoxylin and eosin (H&E) staining was conducted according to standard procedures. Thermal damage is manifested as blanching and thermal cauterization of superficial blood vessels[4]. We checked that the H&E staining images for blanching as a sign of thermal damage. The H&E stain images were obtained with stimulation intensities at 100 Hz with repeated optical stimulations (Figs. S4**a** and **b**). No blanched region was observed. Therefore, the insertion of the optical nerve cuff into the body and the heat resulting from the cuff are expected to cause almost no damage to the nerves and the circumferential muscles.

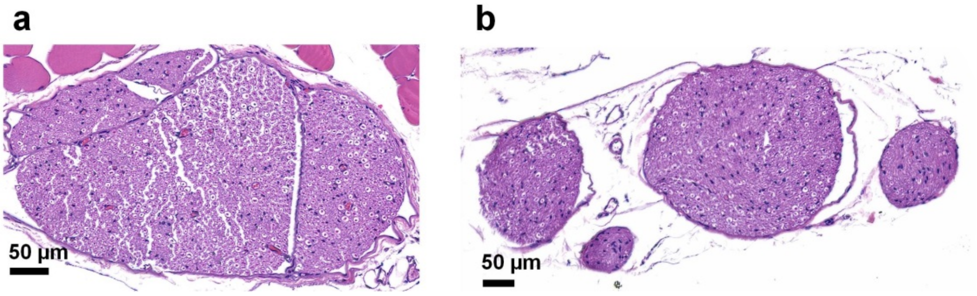

Fig. S4. (a) H&E staining image of the sciatic nerve during a 100 Hz repeated optical stimulation. (b) H&E staining image of the sciatic nerve during a 10 Hz repeated optical stimulation using the proposed optical nerve cuff electrode.

References

[1] Andreasen, L. N. S., Struijk J. J. & Lawrence S Measurement of the performance of nerve cuff electrodes for recording *Med. Biol. Eng. Comput.* **38**, 447–453 (2000).

[2] Triantis, I. F. & Demosthenous, A. Tripolar-cuff deviation from ideal model: assessment by bioelectric field simulations and saline-bath experiments *Med. Eng. Phys.* **30**, 550–562 (2008).

[3] Chu, J. U. *et al.* Improvement of signal-to-interference ratio and signal-to-noise ratio in nerve cuff electrode systems. *Physiol Meas* **33**, 943–967, (2012).
[4] Cherov, M. M, Chen, G. & Roe, A. W. Histological Assessment of Thermal Damage in the Brain following Infrared Neural Stimulation. *Brain Stimul.* **7**(3) 476–482 (2014).
